# Algunos recuerdos y posibles olvidos de mi trayectoria antropológica

**DOI:** 10.18294/sc.2023.4437

**Published:** 2023-04-17

**Authors:** Eduardo L. Menéndez

**Affiliations:** 1 Doctor en Ciencias Antropológicas. Doctor Honoris Causa, Universitat Rovira i Virgili, Catalunia; Universidad Nacional de Rosario, Argentina; Universidad Nacional de Lanús, Argentina. Profesor-investigador emérito, Centro de Investigaciones y Estudios Superiores en Antropología Social (CIESAS), México. emenendez1@yahoo.com.mx Centro de Investigaciones y Estudios Superiores en Antropología Social Centro de Investigaciones y Estudios Superiores en Antropología Social (CIESAS) Mexico emenendez1@yahoo.com.mx; Universitat Rovira i Virgili Catalunia España; Universidad Nacional de Rosario Argentina; Universidad Nacional de Lanús Argentina

**Keywords:** Antropología Médica, Proceso Salud-Enfermedad, Medical Anthropology, Health-Disease Process

## Abstract

Este texto reproduce el discurso pronunciado por Eduardo L. Menéndez, en el marco de la entrega del título de doctor honoris causa, otorgado por la Universidad Nacional de Lanús, el 27 de marzo de 2023. En su discurso recupera su infancia y su trayectoria académica en Argentina, previa a su exilio en México en 1976, y recorre varios procesos que, por diferentes circunstancias, en forma consciente e inconsciente, orientaron su campo de estudio y contextualizan sus explicaciones teóricas.

## Abstract (en)

This text is a transcription of the speech delivered by Eduardo L. Menéndez at the ceremony in which he was conferred a Doctorate Honoris Causa by the Universidad Nacional de Lanús on March 27, 2023. The speech covers aspects of his youth and academic career in Argentina, prior to his exile in Mexico in 1976, and reflects on various processes that due to a number of circumstances consciously or unconsciously oriented his field of inquiry and contextualize his theoretical contributions.

**Figure:**
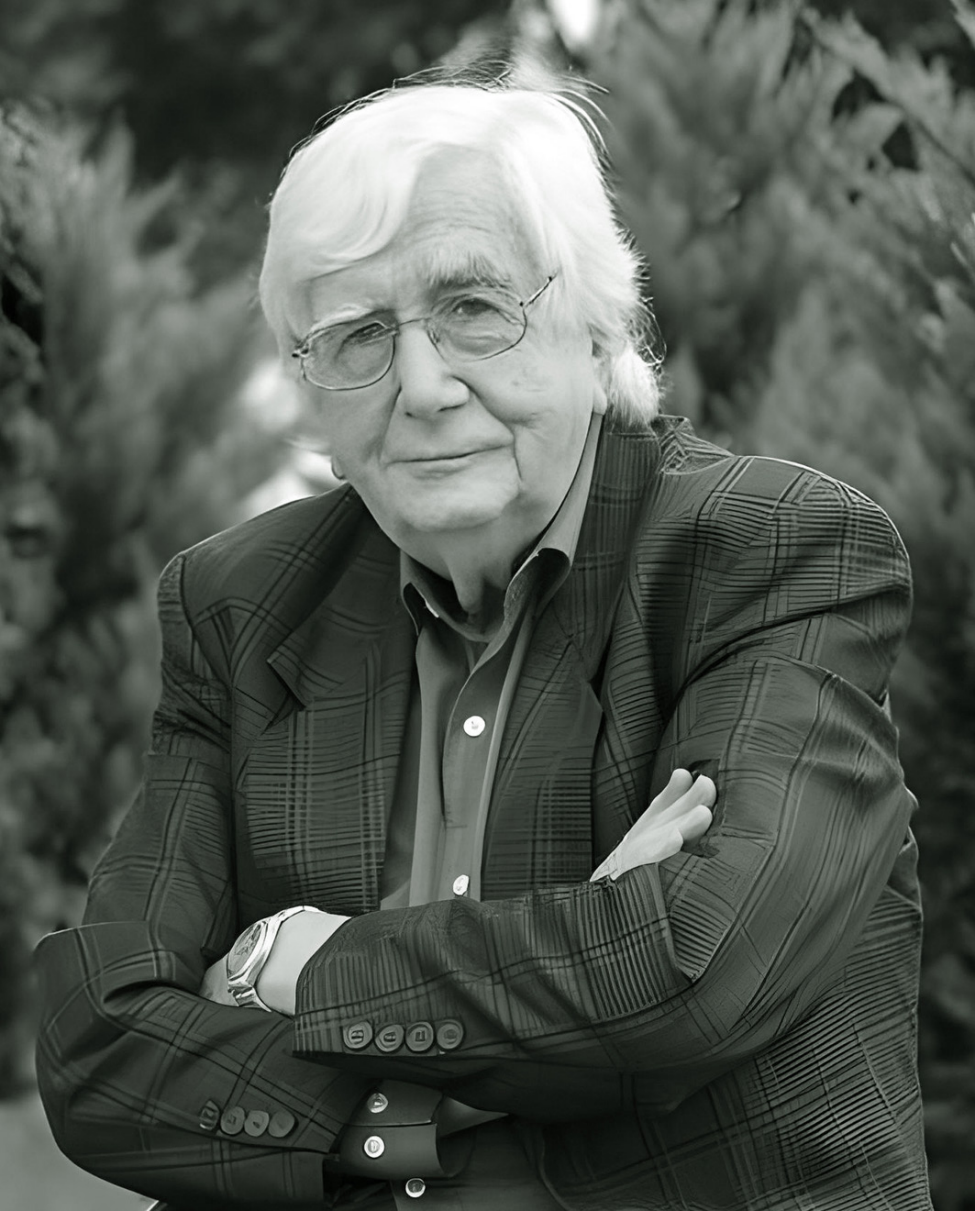
Eduardo L. Menéndez, doctor honoris causa por la Universidad Nacional de Lanús, 2023

Buenas tardes a todas las personas presentes y a las que están conectadas digitalmente. En principio agradezco a la Universidad Nacional de Lanús la entrega del doctorado *honoris causa* y, en particular, agradezco el papel del Instituto de Salud Colectiva, especialmente, del Dr. Hugo Spinelli, quien desde hace más de treinta años ha apoyado y difundido gran parte de las ideas que he generado respecto de los procesos y problemas de salud/enfermedad, y con quien me une una profunda amistad. Y, por último, agradezco la aceptación a hablar sobre mi trabajo a varios especialistas a los que no solo reconozco intelectualmente, sino a los que aprecio afectivamente. 

Señalo que voy a leer mi texto para no desbandarme demasiado, y aclaro que yo pensaba hablar unos 10 o 15 minutos, pero el Dr. Spinelli me pidió que hablara tres cuartos de hora, por lo cual espero que lo que presente no parezca demasiado narcisista, ni una ocasión para el autoelogio.

En esta presentación me referiré sobre todo a mi trayectoria académica, y solo en algún momento -si es que lo hago- a mi trayectoria social y política, pese a que en 1976 tuve que irme de mi país para exiliarme en México, pensando que iba a ser un viaje de ida y vuelta, donde el exilio en México duraría no más de dos años, pero que se convirtió en una estadía definitiva. Yo -como muchxs compañerxs exiliadxs- pensaba estar no más de un par de años fuera de Argentina, tanto fue así que mi familia y yo compramos pasajes de avión de ida y vuelta. Esta actitud tenía que ver con que casi desde la infancia habíamos convivido con varios golpes militares que, luego de una primera etapa dictatorial, reducían poco a poco sus controles, hasta volver a lo que llamamos vida democrática. Pero eso no ocurrió, y se convirtió en el golpe militar más sanguinario, cruel y extenso, por lo menos de los siglos XX y XXI.

Como ya dije, lo dominante en lo que presentaré refiere a mi trayectoria académica articulada a veces con mi vida cotidiana, focalizado en mi trabajo cuando vivía en Argentina. Y si bien hablaré de aspectos que ya están registrados en varias de mis publicaciones, sin embargo, hay algunos procesos que presento por primera vez, y que en su mayoría tienen que ver con cómo manejo teórica e ideológicamente los procesos a estudiar. 

Justamente, uno de los procesos sobre el que casi no he escrito ni hablado tiene que ver con que, a partir de cierto momento, me pregunté en México por qué me había dedicado a lo que llamamos antropología médica; pero no solo referido a aspectos teóricos, prácticos, académicos y políticos, sino a aspectos que tuvieran que ver con mi vida más o menos privada. En términos académicos y políticos yo me había ido acercando a los procesos de salud/enfermedad por dos razones que se fueron precisando cada vez más. Primero, porque era uno de los principales indicadores no solo de las diferencias de clase, sino de las consecuencias negativas en la vida de las clases subalternas, dado que dichas clases y, especialmente, los pueblos originarios tenían y siguen teniendo las más altas tasas de mortalidad general en los diferentes grupos etarios, así como la menor esperanza de vida. Y, segundo, porque a través de los procesos de salud/enfermedad/atención y prevención pueden observarse con mayor transparencia y objetividad, por lo menos una parte de los procesos sociales, económico-políticos e ideológico-culturales centrales de nuestras sociedades.

Considero que en mi vida hubo varios procesos que, por diferentes circunstancias, en forma consciente e inconsciente, me orientaron hacia mi campo de estudio actual y contextualizan mis explicaciones teóricas. A continuación, voy a enumerar algunos de estos procesos.

Tengo un origen de clase baja, mi padre era obrero y mi madre era costurera, y viví mi infancia en barrios de clase baja, en los cuales todas las chicas y los chicos que conocía nos enfermábamos especialmente de mal de ojo y de empacho, pero también de otras enfermedades llamadas por lxs antropólogxs tradicionales, aunque para nosotros y nuestras familias no eran para nada tradicionales. Y eso ocurría en la ciudad que, en mi infancia, tenía la mayor población de América Latina, y el mayor desarrollo industrial y de servicios.

Todas estas enfermedades suponían la aplicación de tratamientos tradicionales que, en el caso del mal de ojo, implicaban procesos de eficacia simbólica que, en mi caso y durante por lo menos dos años, condujeron a que me lo aplicaran casi cada mes, dado que yo era un chico gordito y con apariencia sana, y por eso me ojeaban con cierta reincidencia, al igual que a mi hermana, pero en el caso de ella porque era muy bonita. Junto con esto, mi abuelo calabrés, mis tías y mi madre nos narraban toda una serie de historias mágicas, pero también realistas del campesinado del sur de Italia. Mi padre y mis tías asturianas nos relataban usos y costumbres, así como antiguos romances astures que también tenían que ver con procesos de salud/enfermedad en términos de desgracias y de curas más o menos mágicas. 

Es decir que las enfermedades y tratamientos tradicionales -obviamente con las enfermedades y tratamientos alopáticos- eran parte de mi vida cotidiana infantil. No olvidemos que, en el caso de América Latina, y de México en particular, la medicina tradicional constituye hasta ahora el principal campo de estudio de la antropología médica, pero que se estudia casi exclusivamente en los pueblos originarios. Por eso cuando comencé a especializarme en esta disciplina, primero en Argentina y, sobre todo, en México, me confronté con posiciones dominantes que veían las enfermedades y los tratamientos tradicionales como parte intrínseca de las culturas nativas, que las diferenciaban de los grupos no nativos, y sobre todo de las medicinas occidentales. Lo que para mí contrastaba fuertemente con mi propia experiencia porque, además, varias de las principales enfermedades tradicionales de los pueblos originarios eran las mismas no solo de mi infancia de clase baja urbana, sino también de las principales enfermedades tradicionales de países como Italia y España. Lo que me llevó a formular interpretaciones teóricas que se diferenciaban fuertemente de las dominantes, y estando algunas de ellas publicadas en la revista *Salud Colectiva*, editada por el Instituto de Salud Colectiva.

Un segundo aspecto muy diferente tiene que ver con un hecho ocurrido durante el servicio militar que hice en el hospital de campo de mayo con la categoría de soldado camillero. Una tarde que yo estaba de guardia, junto con el Dr. Etchegoyen, vinieron de la escuela de suboficiales a pedirnos que fuéramos hacia allí, porque un estudiante se había suicidado, pegándose un tiro. Cuando llegamos, el joven estaba tendido en una plancha de cemento y sus padres, que eran santiagueños, le rogaban al cura de la escuela de suboficiales que permitiera que su hijo fuera enterrado en camposanto, a lo que el cura se negaba, dado que la iglesia católica prohíbe que un suicida sea sepultado en un área cristianizada. Yo sabía eso porque hasta los 14 o 15 años fui católico, y en algún lugar lo había escuchado o leído, pero ahora dicha prohibición se me aparecía como un hecho concreto: tenía delante un cadáver producto de un suicidio, padres rogando y un sacerdote negando. Además, en mi recuerdo, para los padres parecía tener más importancia que el hijo no fuera enterrado en camposanto, que el hecho de que estuviera muerto.

Y esto tuvo que ver con varias cuestiones que fui desarrollando ulteriormente. La primera, la relación entre los procesos de salud/enfermedad y el campo religioso en términos conflictivos; la segunda, la importancia de la observación directa de los procesos, y asumir que gran parte de lo que sabemos sobre nuestra propia cultura es un conocimiento liviano y sin profundidad que se actualiza ante situaciones puntuales; y, la tercera, registrar en forma casi inadmisible que, mientras un suicida no debe ser enterrado en camposanto, sin embargo, un multihomicida no tiene ningún problema para ser sepultado en tierra sagrada según la religión católica. Estos y otros procesos condujeron a que, años más tarde, elaborara un taller sobre el suicidio para estudiantes de Antropología, y que -y lo subrayo- todavía lo sigo aplicando, y no solo a estudiantes de Antropología, como ocurre en el caso de los seminarios que imparto en la Universidad Nacional de Lanús.

Mi preocupación por el suicidio me llevó a leer el texto de Durkheim, que sigue siendo para mí, más allá de sus incorrecciones empíricas, el principal texto teórico y metodológico que se ha escrito sobre el suicidio. Más aún, es el que ha generado más interpretaciones sobre dicho problema, dado que Durkheim se ha caracterizado por su orientación y capacidad interpretativa, pese a ser considerado un “positivista”. Y este es un aspecto que me interesa subrayar: ninguna de las alumnas y los alumnos que he tenido en mis seminarios había leído a Durkheim, y en la mayoría dominaba la idea de que era un simple positivista. Y este aspecto me interesa rescatarlo porque en gran medida expresa algo dominante en las ciencias duras desde hace años, y que cada vez se ha filtrado con más fuerza en la antropología. Y me refiero a que un texto considerado “viejo” ya no debe ser leído; como ustedes saben, hasta hace poco las ciencias duras consideraban “viejo” un texto publicado hace diez años; pero en la actualidad es “viejo” un texto publicado hace cinco años. Esta tendencia puede ser nefasta para el desarrollo de nuestras disciplinas.

El tercer caso tiene que ver con la tuberculosis broncopulmonar que contraje entre los 22 y 23 años, y que me mantuvo en cama durante nueve meses, y de la cual pude salvarme de morir por la aplicación de un tratamiento basado en estreptomicina y nicotibina. Dicho padecer supuso tener una profunda experiencia de muerte ya que, en los primeros meses, dado el diagnóstico médico, suponía que iba a morir, aunque lo vivía tranquila y literariamente, lo que me permitió reflexionar sobre qué era la muerte individual, y la necesidad o no de vivir. Fue además la primera vez que asumí que la vida es rutina y repetición, frente a lo que elaboré una solución elitista basada, sobre todo, en Nietzsche y Sartre, que más tarde reelaboré. Por otra parte, ese tiempo en cama fue en gran medida la base de mi “capital cultural”, pues durante nueve meses no tenía otra cosa que hacer que leer.

Y un último caso tiene que ver con mi forma de describir y analizar los procesos que estudio, y me refiero al hecho de que entre los 17 y los 23 años yo pertenecía a un grupo literario y escribía poesía, que nunca intenté publicar, pero que me llevó más tarde a que al escribir sobre los procesos que estudiaba de salud/enfermedad lo hiciera en forma fría y distante y, además, a rechazar todo trabajo sobre procesos de salud/enfermedad que escribiera más o menos literariamente. Solo en mis últimos años me he permitido redactar algunos textos en los que hay algunos apartados más o menos literarios, de los cuales el más notorio es uno publicado hace dos años en la *Revista Latinoamericana de Antropología del Trabajo*. Reconozco que esta actitud no fue ni es correcta, aunque, sin embargo, algunos trabajos académicos que pretenden ser literarios confirman mi temprana decisión. 

Hay otros procesos que tienen que ver con mi formación como antropólogo y con mis primeras actividades de investigación. Aclaro que comencé estudiando historia antes que antropología, y casi al final de la carrera formé parte de un seminario sobre historia de las mentalidades que coordinaba José Luis Romero, que duró tres años, y en el cual siete personas discutíamos materiales correspondientes a la alta y tardía edad media y al renacimiento de países europeos; una parte de los cuales trataban sobre procesos de salud/enfermedad, a los cuales la mayoría de los analistas franceses aplicaban interpretaciones de tipo durkheimiana. Esta formación histórica previa y, especialmente, el citado seminario, me llevaron a que frente a cualquier proceso de salud/enfermedad, tratara de aplicar la dimensión histórica, pero no pensada en trayectorias individuales, sino en trayectorias sociales. Considero que casi todo proceso de salud/enfermedad debe ser pensado en términos de historicidad, aunque más no sea a nivel reflexivo.

Ya casi al final de mi carrera, en un seminario que impartía Rodríguez Bustamante sobre teoría sociológica, que incluía a Marx, le pregunté cómo podía hacer para tener una visión crítica del marxismo y él me dijo: “lea a los fascistas”. Lo que me quiso decir provocativamente Rodríguez Bustamante es algo que sigo aplicando hasta la actualidad y es que, respecto de los problemas que analizo a través de cierta teoría e interpretaciones, necesito leer textos que apoyen dicha teoría e interpretaciones, pero también textos que las critiquen en profundidad e incluso sesgadamente. Esto lo considero cada vez más necesario, dado el incremento constante de teorías cada vez más ideologizadas; aclaro que no cuestiono dicha ideologización, sino la necesidad de objetivarlas y manejarlas. Creo que, por lo menos una parte de los antropólogos actuales no tiene idea de que quienes están más de acuerdo con las teorías ideologizadas son los teóricos de extrema derecha y, especialmente, los neonazis.

Yo era un joven antropólogo recién graduado cuando recibí una invitación para conversar con el director del instituto de cancerología que quedaba en Agronomía. Fui y el Dr. Schavelzon me informó que desde hacía varios años venía observando que toda una serie de pacientes con cáncer terminal acudían a la capilla católica a rezar, y que no solo iban católicos, sino personas de otras religiones. Me dijo, además, que había observado que estas personas que rezaban tenían una mayor esperanza de vida, solicitándome que hiciera una investigación sobre dicho proceso, a lo que respondí honestamente que no contaba con los saberes y experiencia suficientes para hacerlo. Considero que no solo esto operó, sino también mi miedo personal a trabajar con temas y sujetos que me parecían no solo complejos, sino próximos a morir. Pero uno de los resultados de la conversación con el Dr. Schavelzon fue asumir la relevancia que un importante especialista biomédico le daba a los aspectos religiosos y simbólicos en los procesos de salud/enfermedad, lo que no suele ser reconocido por lxs antropólogxs, al menos, latinoamericanxs. Por el contrario, domina la idea de que los ignoran y/o rechazan.

**Figure:**
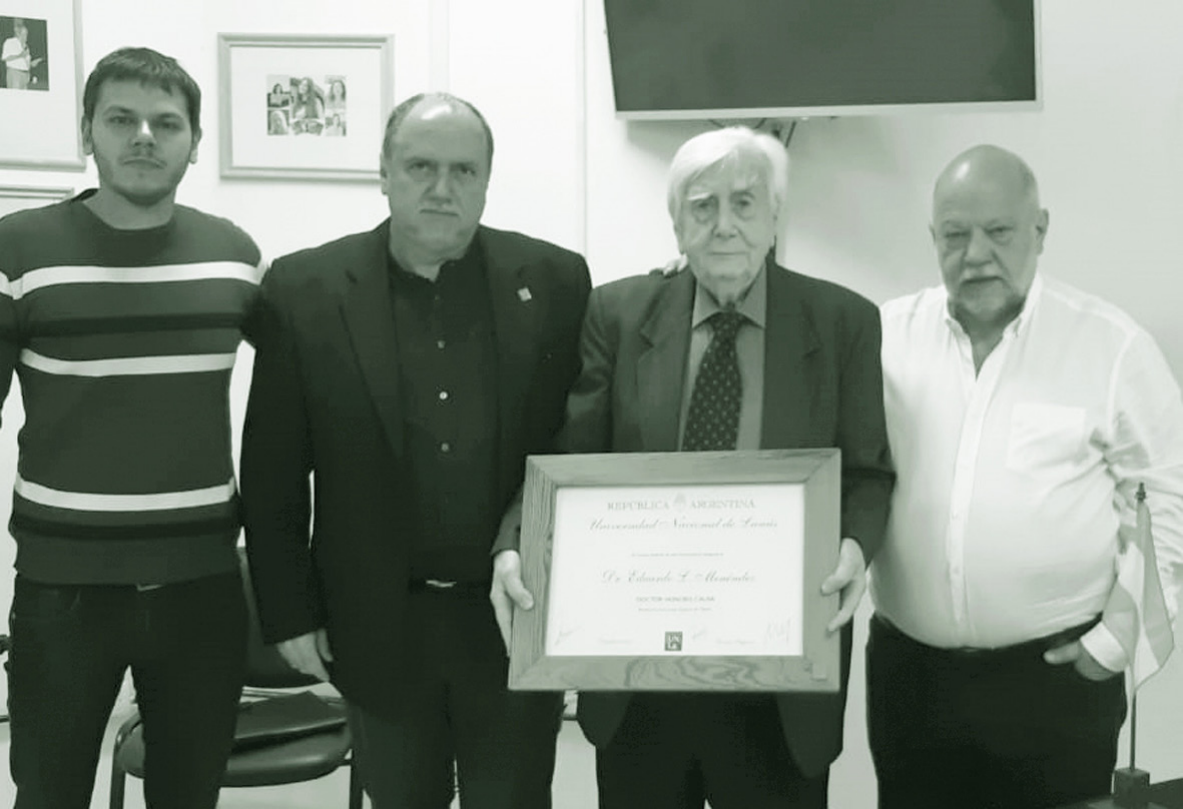
Eduardo L. Menéndez con el diploma de doctor honoris causa. En representación de la Universidad Nacional de Lanús, lo acompañan, a la izquierda de la foto, Daniel Bozzani (vicerrector) y Marcos Mele (secretario de Investigación y Posgrado) y, a su derecha, Hugo Spinelli (director del Instituto de Salud Colectiva). 27 de marzo de 2023.

Dicha importancia se verificó unos cinco años después, cuando el Dr. José Bleger, considerado uno de los principales psicoanalistas argentinos, me citó para conversar sobre procesos de salud mental y cómo los veía desde mis perspectivas antropológicas. A partir de ese intercambio de ideas, me propuso integrar un grupo que él coordinaba, constituido por diez psicoanalistas, y que analizaban problemas críticos de salud mental en nuestro país, lo que me permitió nuevamente observar la importancia que les daban a los aspectos culturales y simbólicos, por lo menos ciertos especialistas.

Ahora bien, este reconocimiento no solo aparecía en las concepciones de estos importantes médicos argentinos, sino que, en función de mis relaciones con las instituciones médicas y de mis investigaciones tanto en Argentina como en México, observé que la mayoría de los médicos, por lo menos hasta finales de la década de 1990, sabían y reconocían la existencia de estos procesos, aun cuando la mayoría no trabajara con ellos. En gran medida, esto se daba, en primer lugar, porque no sabían muy bien qué hacer con ellos y, en segundo lugar, porque los veían como contradictorios respecto del saber biomédico que aprendían y ejercían. Estas reflexiones fueron parte de mi formulación del modelo médico hegemónico, y que desarrollé sobre todo en mis estudios sobre saber médico y alcoholismo realizados en México.

Mi primer estudio, que implicó trabajo de campo socioantropológico, fue sobre un juego infantil: la rayuela. Lo hice en escuelas de la Ciudad de Buenos Aires, observando y entrevistando a niños que jugaban este juego. El trabajo se hacía a partir de un marco teórico que consideraba que casi todos los juegos infantiles constituyen continuidades y reformulaciones de ciertos ritos sociales básicos que perdieron vigencia y se convirtieron en juegos. En el caso de la rayuela, sería originalmente un rito de iniciación en el que el sujeto va de la tierra al cielo saltando a pie cojo, como Edipo. Y lo más importante que encontré es que obviamente los niños no tenían la menor idea de que lo que hacían como juego fue previamente un ritual social; ni sabían qué significaba ir de la tierra al cielo a través de la rayuela. Pero este hecho, según el cual los sujetos -especialmente respecto de ciertos procesos- no tienen mucha noción qué significa realmente lo que hacen, no solo lo encontré en los juegos infantiles sino en usos y costumbres médicas de grupos tradicionales. Y así, por ejemplo, en un ritual curativo realizado en comunidades yucatecas, los sujetos no tenían idea de lo que significaba el discurso que tenían que decir en maya. Lo sustantivo en ellos era la eficacia lograda, y no entender lo que ocurría. 

Las dos investigaciones más importantes en las que inicialmente trabajé como antropólogo no tuvieron que ver centralmente con los procesos de salud/enfermedad, aun cuando en esas investigaciones se estudiaron procesos que me llevaron a profundizarlos luego. La primera de ellas, fue en una comunidad de unos 10.000 habitantes en la provincia de Entre Ríos, y trató sobre los procesos de migración y asentamiento de población italiana y española desde finales del siglo XIX hasta 1960. En este estudio apareció un hecho que iba a ser parte importante de mis objetivos etnográficos y teóricos; y me refiero a que encontré que tanto los migrantes italianos como los españoles habían constituido desde finales del siglo XIX sociedades de socorros mutuos, según las cuales cada familia migrante debía aportar una cierta cantidad de dinero para tres actividades básicas: atender enfermedades graves, atender los gastos generados por la muerte de un sujeto, y ayudar a las familias en las que había muerto el sujeto que sostenía a dicha familia. En el caso de la comunidad italiana, había contratado médicos desde el inicio de la sociedad de socorros mutuos, y el primero de ellos fue un famoso médico, pero también antropólogo italiano, preocupado entre otras cosas por los aspectos culturales de la salud/enfermedad, y quien se llamaba Paolo Mantegazza.

Ahora bien, registré esta actitud colectiva de ambos grupos migrantes como parte de la autoatención en términos de autogestión social, y observé en forma secundaria los procesos de autoatención que se daban al interior de las familias con que trabajé. Subrayo esto, porque la autoatención más adelante pasaría a ser para mí uno de los principales modelos de atención que opera a nivel de toda sociedad, y que en ciertos conjuntos sociales puede estar relacionadas con procesos de autogestión social. Para mí, en términos más o menos utópicos, esto tenía que ver cada vez más con que las posibilidades futuras de una sociedad equitativa, igualitaria y solucionadora de los problemas básicos de todos sus miembros están en una sociedad de tipo autogestionaria, lo que sigo pensando hasta la actualidad.

El segundo estudio es una investigación interdisciplinaria que dirigí con un economista, y que trató de estudiar el nivel de vida de la población rural de la provincia de Misiones, en la cual se manejaron aunque en forma secundaria datos demográficos, incluidos los de morbimortalidad. Aquí observamos los fuertes déficits de las clases subalternas rurales en todos los campos de la realidad; pero, además, y es lo que me interesa destacar, observamos fuertes procesos racistas, que no solo estaban referidos a grupos indígenas, sino especialmente a población de origen polaco. Y la población polaca era una población caracterizada por su blancura, pese a lo cual era fuertemente racializada, lo cual junto con el antisemitismo que se da también sobre población en su inmensa mayoría blanca, constituyó también parte nuclear de mi marco teórico e ideológico. Esto no es secundario para los procesos de salud/enfermedad, dado que uno de los aspectos que más han registrado los estudiosos de la medicina tradicional -por lo menos en México- es el fuerte racismo que se observa en la relación entre las instituciones médicas y el personal biomédico con relación a la población indígena, lo que los condujo no obstante a manejar una visión racista limitada. Si bien esta orientación antropológica ha cumplido un necesario e importante papel en la denuncia del racismo médico antiindígena, ha tendido a ignorar que el racismo también opera respecto de otros grupos sociales en México, en particular, hacia los afroamericanos. Pero hasta hace muy pocos años, la antropología mexicana solo refería exclusivamente el racismo hacia los pueblos originarios, lo cual supone una vez más el dominio de orientaciones ideológicas, que excluyen otros racismos y limita comprender nuestras realidades.

Ahora bien, el racismo ha sido una preocupación personal que me ha acompañado desde mi juventud hasta la actualidad; mis primeras publicaciones fueron sobre racismo, y varias las organicé hace pocos años para publicar dos libros en México, en los que domina la relación entre racismo y situación colonial. Pero uno de los aspectos que más desarrollé es el que tiene que ver con la presencia del racismo como parte del modelo médico hegemónico, y que no solo se reduce a las investigaciones y acciones médicas nazis, sino a ser parte normalizadas de la medicina estadounidense, británica, francesa, de otros países europeos y, por supuesto, de Latinoamérica. Tempranamente publiqué un texto sobre racismo y violencia científica, que trata sobre estos aspectos, y que todavía sigue siendo consultado en México y Argentina.

Entre ambos estudios, participé en una investigación que se hizo en el Instituto Di Tella que trataba específicamente sobre demandas de atención de padecimientos por la población y las formas de atención posibles en una parte del barrio de Saavedra de la capital federal, y que era coordinado por Esther Hermite y yo, y en el que participaron varios antropólogos argentinos. El estudio fue casi un desastre debido a varias razones, pero especialmente a la existencia de objetivos diferentes en los coordinadores del proyecto. Y si recupero este estudio, no es solo para recordar la existencia de experiencias negativas en mi formación y acción, sino porque aprendí que el desarrollo de un proyecto colectivo implica la posibilidad de diferencias no secundarias que necesitan ser resueltas antes de comenzar la investigación, lo que fue muy útil para experiencias colectivas ulteriores.

Ahora bien, en el transcurso de la trayectoria señalada que se desarrolla entre 1958 y 1968, comencé a recibir demandas de grupos e instituciones psicoanalíticas. Una de las principales fue la demanda de dos destacados psicoanalistas, Gilou y Diego García Reinoso, para participar junto con otra psicoanalista y un filósofo en el análisis de material clínico de pacientes con problemas psicológicos, para proponer interpretaciones de los casos. Poco tiempo después, el psicoanalista Hernán Kesselman me propuso integrar un grupo de estudio en el que participaban cuatro psicoanalistas, un filósofo y yo, para analizar problemas de salud mental y los usos de la violencia desde una perspectiva política e ideológica.

Estas actividades fueron acompañadas por la demanda de seminarios por parte de grupos de psicoanalistas, de los cuales el más significativo fue el que di sobre psicoanálisis e ideología, que culminó con la impartición de cursos sobre salud mental y antropología en el principal servicio de salud mental de la Argentina de aquellos años: el que dirigía el Dr. Mauricio Goldenberg en el Hospital Lanús.

A principios de la década de 1970, el psicoanalista Rubén Efron me invitó a formar parte de un equipo de investigación interdisciplinario en el Centro de salud laboral de la Facultad de Medicina de la Universidad de Buenos Aires, y en el cual participé en estudios realizados con trabajadores ceramistas, con trabajadores mineros, y con choferes de transporte colectivo. El objetivo de todos los estudios era el mismo: detectar cuáles eran los problemas de salud laboral que reconocían los propios trabajadores, y ver con ellos las posibilidades de solucionarlos. Utilizamos una metodología formulada en Italia con enfoque gramsciano, y que se basaba en el punto de vista del trabajador.

El mayor impacto que mantengo hasta la actualidad fue cuando visité por primera vez el sindicato minero y me enteré de que, en Argentina, no se había jubilado nunca un trabajador minero, dado que morían por accidentes, silicosis o neumoconiosis, o eran pensionados por enfermedad durante el transcurso de su vida laboral, sin poder llegar a jubilarse. Y esto lo sabían los mineros, era obvio para ellos que su trabajo generaba enfermedad y muerte; lo que me llevó junto con otros procesos a asumir -como decía Ernest Becker- que la Antropología es, en gran medida, el estudio de lo obvio.

Gran parte de los estudios se hacían solicitados por trabajadores y sindicatos, y dado el tipo de información que producíamos y su uso, comenzaron las amenazas, que se concretaron en la colocación de una bomba de bajo poder en el centro de salud ocupacional, pese a lo cual seguimos trabajando. Pero al poco tiempo pusieron otra bomba de alto poder, que destruyó gran parte del centro y abandonamos nuestras investigaciones. Eran los tiempos de la Alianza Anticomunista Argentina (AAA), también conocida como Triple A, y del desarrollo de la lucha armada, que dieron por resultado el asesinato o la desaparición de varios amigos y, especialmente, de alumnos de la carrera de antropología que yo dirigía en la Universidad Nacional de Mar del Plata.

En los estudios realizados, considero que fui aprendiendo más de lo que aporté y, por supuesto, además de la información y análisis producidos que develaban las consecuencias de las formas de trabajo en la salud de los trabajadores, fui impactado emocionalmente por algunas situaciones particulares. Y la que más me impactó fue generada por una investigación en la que no participé, pues fue realizada por otro equipo sobre la salud física y mental de trabajadores metalmecánicos, especialmente, sobre los trabajadores de la industria automotriz. La investigación evidenció que se estaba dando un proceso de reducción o desaparición de los deseos sexuales de los trabajadores debido al saturnismo que contraían en el proceso productivo. Esta información fue presentada en la Facultad de Medicina, con la participación de los trabajadores, que relataron las consecuencias del saturnismo en su vida cotidiana y, especialmente, en las relaciones sexuales negativas que se construían en torno a sus parejas femeninas.

Es a partir de la participación y trabajo con especialistas en salud mental, así como los estudios sobre salud ocupacional, que alrededor de 1974-1975 comencé a plantearme la cuestión de los modelos de atención y, especialmente, del que denominé *modelo médico hegemónico*. Apliqué la concepción de tipo ideal desarrollada por Max Weber, que ya había aplicado previamente a la producción antropológica, y comencé en forma intermitente a plantear cuáles serían las características estructurales de dicho modelo. Paralelamente, y en función de mi experiencia con psicoanalistas y clínicos, elaboré materiales sobre las relaciones entre antropología y salud mental. Y dicho trabajo fue intermitente, por la situación que atravesaba Argentina, y yo en particular, dado que me habían expulsado de la Universidad Nacional de Mar del Plata y, además, tenía una cierta participación política. Pero pese a la intermitencia elaboré dos textos sobre los dos procesos señalados que publiqué en México al poco tiempo de mi llegada a dicho país.

A pesar de lo que estaba pasando, no pensaba irme de Argentina, aun cuando me habían ido a buscar tres veces a mi casa y, por lo tanto, vivía en otro lugar; y pese a que mi mujer me planteaba el peligro que corríamos, y que yo en parte negaba por la idea que tenía de los golpes militares en Argentina y por otras razones que nunca elaboré. Incluso, continué mi trabajo en forma limitada, dado que quien era director del Departamento de Ciencias de la Educación en Mar del Plata, en el período en que yo dirigía el Departamento de Antropología, había obtenido recursos que le permitían crear un pequeño instituto de ciencias sociales, y me invitó a trabajar en él. Pero cuando aproximadamente al año el cadáver de Guillermo Sablov apareció asesinado en las vías del ferrocarril de la estación Constitución, decidí dejar mi país.

Si bien creo que ya los he señalado, quisiera recuperar tres hechos. El primero tiene que ver con algo que, por lo menos durante la década de 1960 y 1970, caracterizaba la vida intelectual en Argentina, y me refiero al trabajo de reflexión y discusión en grupos de tipo interdisciplinario, y de los cuales rescato el coordinado por José Luis Romero; el organizado por los psicoanalistas Diego y Gilou García Reinoso; el organizado por Hernán Kesselman; el que formamos Ramón Alcalde, Ricardo Piglia, León Rozitchner y yo; el que organicé con antropólogos, sociólogos e historiadores y, especialmente, con Mirta Lischetti y María Rosa Neufeld; y el grupo literario impulsado por Eduardo Prieto. El segundo hecho refiere a la posibilidad de extender el conocimiento más allá del ámbito de los especialistas, y por eso dirigí colecciones de libros en dos editoriales de Argentina, y más tarde en una editorial mexicana, así como participé en la publicación de textos en revistas de difusión.

Y el tercer hecho tiene que ver con mi genealogía intelectual, que es un tanto variada, que publiqué hace años en una revista española, y que no voy a retomar ahora, sino solo señalar que, a partir de los 18 o 19 años, me asumí como marxista, primero en términos teóricos e ideológicos, y luego en términos de participación política. De esa genealogía, que incluye intelectuales estadounidenses, franceses, latinoamericanos y de otros países, rescato dos intelectuales no antropólogos, pero que me influenciaron hasta la actualidad, y me refiero a Antonio Gramsci y a Frantz Fanon. De Gramsci asumí entre otras su propuesta de las relaciones de hegemonía/subalternidad/contrahegemonía, así como la interpretación y el uso de la cultura cotidiana en términos políticos e ideológicos; y recordemos que los procesos de salud/enfermedad son parte básica de la cultura y vida cotidiana de toda sociedad. Y de Fanon, que no olvidemos que era psiquiatra, aprendí cómo la sociedad puede generar enfermedades en función de las situaciones en las que sufren los sujetos; y cómo los sujetos subalternos pueden manejar una cara profunda propia y otra construida para el colonizador, como mecanismo descolonizador. Y de ambos confirmé la necesidad de estudiar y trabajar con los sectores subalternos en términos relacionales, y no en términos individuales.

Llegué a México una semana después del golpe militar dado en Argentina, es decir, el 1 de abril de 1976, y decidí inicialmente, por varias razones, no buscar trabajo en instituciones antropológicas. Por eso, y por mis objetivos de trabajo, decidí trabajar en la Escuela de Salud Pública de México, en el departamento de sociología que dirigía un médico llamado Ricardo Loewe, quien se convertiría en uno de mis mejores amigos hasta su muerte, a finales del año pasado.

En dicha escuela, di seminarios de posgrado, dirigí tesis, y participé en pequeñas investigaciones, para luego desarrollar un proyecto de investigación más o menos integral. El curriculum de esta escuela había sido generado a partir del propuesto por la Escuela de Salud Pública de la Universidad Johns Hopkins, que tenía como una de sus bases desarrollar la formación del personal de salud pública a través de experiencias directas en campo. De allí que, primero, los alumnos debían tener una estadía en campo de tres semanas para estudiar el sistema de salud que se aplicaba a nivel de un estado y, segundo, un grupo de alrededor de diez alumnos debía participar en un estudio de comunidad que duraba cuatro semanas y que era en gran medida de tipo antropológico. Estas prácticas ya no son parte en la actualidad de la formación de los salubristas mexicanos, en las que participaron varios de los principales antropólogos como Gonzalo Aguirre Beltrán y Guillermo Bonfil Batalla.

Fue luego de dos años de mi estadía en México que decidí relacionarme con instituciones antropológicas, por lo que junto con Ricardo Loewe le propusimos a Bonfil Batalla, entonces director del CIS-INAH (luego CIESAS), realizar dos investigaciones sobre procesos de salud/enfermedad en dos estados del país: Michoacán y Yucatán. Yo trabajé en Yucatán.

Pero mi plática llega hasta aquí, no incluyendo mi trabajo en México, que es donde más desarrolle mis estudios y reflexiones sobre los procesos de salud/enfermedad. Así como también mi trabajo en la Universitat Rovira i Virgili de Tarragona, que fue decisivo para la convalidación de varias de mis propuestas. No obstante, quiero señalar que, a partir de 1984 y 1985 comencé a venir a Argentina casi todos los años para dar seminarios de antropología y salud en diversos ámbitos, y en uno de eso viajes fui invitado por el Dr. Spinelli a participar en discusiones sobre a atención primaria, que condujeron a anudar una amistad que llega hasta hoy. Por eso, para concluir, agradezco una vez más a la Universidad Nacional de Lanús la entrega del doctorado *honoris causa*.

Por ahora nada más, y muchas gracias por haberme escuchado.

